# Primary and Recurrent Repair of Incisional Hernia Based on Biomechanical Considerations to Avoid Mesh-Related Complications

**DOI:** 10.3389/fsurg.2021.764470

**Published:** 2021-12-15

**Authors:** Regine Nessel, Thorsten Löffler, Johannes Rinn, Philipp Lösel, Samuel Voss, Vincent Heuveline, Matthias Vollmer, Johannes Görich, Yannique-Maximilian Ludwig, Luai Al-Hileh, Friedrich Kallinowski

**Affiliations:** ^1^General, Visceral and Pediatric Surgery, Klinikum Am Gesundbrunnen, Heilbronn, Germany; ^2^General and Visceral Surgery, Gesundheitszentrum Rhein-Neckar Hospital Eberbach, Eberbach, Germany; ^3^General and Visceral Surgery, Kreiskrankenhaus Bergstrasse Hospital Bergstrasse, Heppenheim, Germany; ^4^Engineering Mathematics and Computing Lab, Interdisciplinary Center for Scientific Computing, Heidelberg, Germany; ^5^Laboratory of Fluid Dynamics and Technical Flows, Otto-von-Guericke University Magdeburg, Magdeburg, Germany; ^6^Biomechanics, Hamburg University of Technology, Hamburg, Germany; ^7^Radiological Center, Eberbach, Germany; ^8^General, Visceral and Transplantation Surgery, University Hospital Heidelberg, Heidelberg, Germany

**Keywords:** bench test, computerized tomography, incisional hernia, GRIP, hernia repair, hernia repair mesh, CRIP

## Abstract

**Aim:** Mechanical principles successfully guide the construction of polymer material composites in engineering. Since the abdominal wall is a polymer composite augmented with a textile during incisional hernia repair we ask: can incisional hernia be repaired safely and durably based on biomechanical principles?

**Material and Methods:** Repair materials were assessed on a self-built bench test using pulse loads to elude influences on the reconstruction of the abdominal wall. Tissue elasticity was analyzed preoperatively as needed with computed tomography at rest and during Valsalva's maneuver. Preoperatively, the critical retention force of the reconstruction to pulse loads was calculated and a biomechanically durable repair was designed based on the needs of the individual patient. Intraoperatively, the design was adjusted as needed. Hernia meshes with high grip factors (Progrip^®^, Dahlhausen^®^ Cicat) were used for the repairs. Mesh sizes, fixation elements and reconstructive details were oriented on the biomechanical design. All patients recieved single-shot antibiosis. Patients were discharged after full ambulation was achieved.

**Results:** A total of 163 patients (82 males and 81 females) were treated for incisional hernia in four hospitals by ten surgeons. Primary hernia was repaired in 119 patients. Recurrent hernia was operated on in 44 cases. Recurrent hernia was significantly larger (median 161 cm^2^ vs. 78 cm^2^; u-test: *p* = 0.00714). Re-do surgery took significantly longer (median 229 min vs. 150 min; *p* < 0.00001) since recurrent disease required more often transversus abdominis release (70% vs. 47%). GRIP tended to be higher in recurrent repair (*p* = 0.01828). Complication rates (15%) and hospital stay were the same (6 vs. 6 days; *p* = 0.28462). After 1 year, no recurrence was detected in either group. Pain levels were equally low in both primary and recurrent hernia repairs (median NAS = 0 in both groups at rest and under load, *p* = 0.88866).

**Conclusion:** Incisional hernia can safely and durably be repaired based on biomechanical principles both in primary and recurrent disease. The GRIP concept provides a base for the application of biomechanical principles in incisional hernia repair.

## Introduction

Incisional hernia is a frequent side-effect of major surgery. Herniated abdominal walls are augmented with textile mesh but mesh-related complications are claimed. Recurrence and pain are common after incisional hernia repair reflecting a lack of mechanical strength at the mesh-tissue interface. Weak bonds between meshes and tissues can be mechanically overloaded. As a result, an occult fascial dehiscence occurs (1). Since non-crosslinked collagen stretches but cannot gain strength, this dehiscence will not heal (2). A continuously overburdened tissue-mesh compound manifests later as a recurrent hernia (3, 4). A recurrent hernia causes pain, loss of work and poverty.

Most repair materials are currently tested outside of a compound structure (5). Load-bearing soft tissues of various individuals exhibit different strain levels (6). An intimate connection of properly dimensioned materials is a prerequisite for a durable hernia repair. Thus, a failed repair can be considered as an unfavorable selection of repair materials not adjusted to the tissues.

Human abdominal walls are stretched with every breath and with each body motion. Durable incisional hernia repair requires materials tested with low cyclic loading in a compound structure. Such materials became available with a bench test using pulse loads (7, 8). The next task was the adaptation of the materials to the individual needs. The individual abdominal wall was assessed with computed tomography of the abdomen with Valsalva's maneuver (8, 9).

Here, we report for the first time primary and recurrent incisional hernias treated according to the GRIP concept. Techniques were analyzed on a biomechanical basis. The needs for a durable repair were defined. Relevant biomechanical parameters were calculated. The resistance gained to withstand repeated impacts was determined. The process to design a repair is highly flexible. It was adapted to any challenge posed by more than 160 clinical situations so far.

## Materials and Methods

### Clinically Relevant Biomechanical Data Derived From Bench Tests With Pulse Loads of Mesh-Tissue Compounds

Biomechanical properties of mesh-tissue compounds derived from low cyclic loading are critically underreported (10). Surgeons get almost no information on biomechanical aspects of hernia meshes. However, such information is necessary to design a durable hernia repair (11). A mismatch of the mechanical properties of meshes and tissues leads to a failure of the mesh-tissue compound upon cyclic load (12). Only about 30% of the package labels contain any information on the mesh biomechanics in a mesh tissue compound. This is in sharp contrast to material sciences where cyclic loading has become increasingly important as a tool for characterization of materials and design of engineered structures (13). Meshes and fixation materials have to be analyzed with regards to the loading mode, the stress-strain relationship and other entities (14). In engineering sciences, a multitude of methods and coefficients is available to characterize properties, stress states and load limits of materials and compounds (15).

Our group developed a bench test for cyclic loading as a model of incisional hernia repair and called it dynamic intermittent strain (DIS; 7–9). The bench test can be run with a variation of the pulse load (11). Mesh coefficients describe the tackiness of the meshes and their resistance to dynamic stiction for 12 meshes. A 14-fold variation is found so far. Mesh coefficients can vary according to testing under dry and wet conditions and related to the lubricant used by up to 75%. The mesh anisotropy should be considered when determining the preferred direction and can alter the retention force by 50% (7). As test tissue, porcine abdominal wall and bovine flank are characterized with a four-fold GRIP variation. In the human situation, the tissue quality varies about 18-fold and should be analyzed in critical cases such as complex or recurrent repair (11). The mesh position as retromuscular, sublay and onlay varies the dynamic stiction by about two-fold. Fixation elements can be divided as strong (sutures), intermediate (tacks), and weak (glue) bonds. Here, the coefficients vary six-fold.

Mesh coefficients are known for all DIS classes. DIS class A meshes are Progrip^®^ (coefficent: 1.44) and Dynamesh^®^ Cicat (coefficient: 1.0). DIS class B meshes are Ultrapro^®^ (coefficient: 0.25), Optilene^®^ (coefficient 0.4) and Ultrapro^®^ Advanced (coefficient: 0.5). Adhesix^®^ borders on DIS class C (coefficient: 0.1). Position coefficients for onlay repairs are 0.5, for sublay/retromuscular 1.0 and for underlay/IPOM 0.9. Fixation coefficients are known for strong (sutures; coefficient: 0.5 per bite) and for weaker fixation techniques such as most tacks (coefficient: 0.3 per tack). As an exemption, Securestrap^®^ must be classified as strong (coefficient: 0.5 per tack). Glues such as Glubran^®^ or Tisseel^®^ can be calculated per area in the case of flat gluing or per spot (width: 8 mm). Care has to be taken for a minimal layer thickness of 1 mm. Sufficient time and temperatures at the glued surfaces above 35 °C should be allowed for the development of adequate adhesive power since binding is a time- and temperature-dependent process for glues. Two adhesive points are equivalent to one square centimeter (coefficient per spot or per half cm^2^: 0.15). From these coefficients, GRIP values can be calculated for the design of a hernia repair.

The data can be summarized for the individual reconstruction into a critical retention force to be surpassed (CRIP). During and after the reconstruction, the gained resistance to impacts related to pressure (GRIP) can be calculated. The patient data are collected in the Herniamed^®^/Stronghold registry. In a first approach, we found no recurrence and low pain levels in the first patients (8, 9). Here, we report a larger patient base divided into primaries and recurrences treated on a biomechanical basis.

### CT Abdomen at Rest and During Valsalva's Maneuver–Example of the Procedure for One Patient

**Figure 1:**
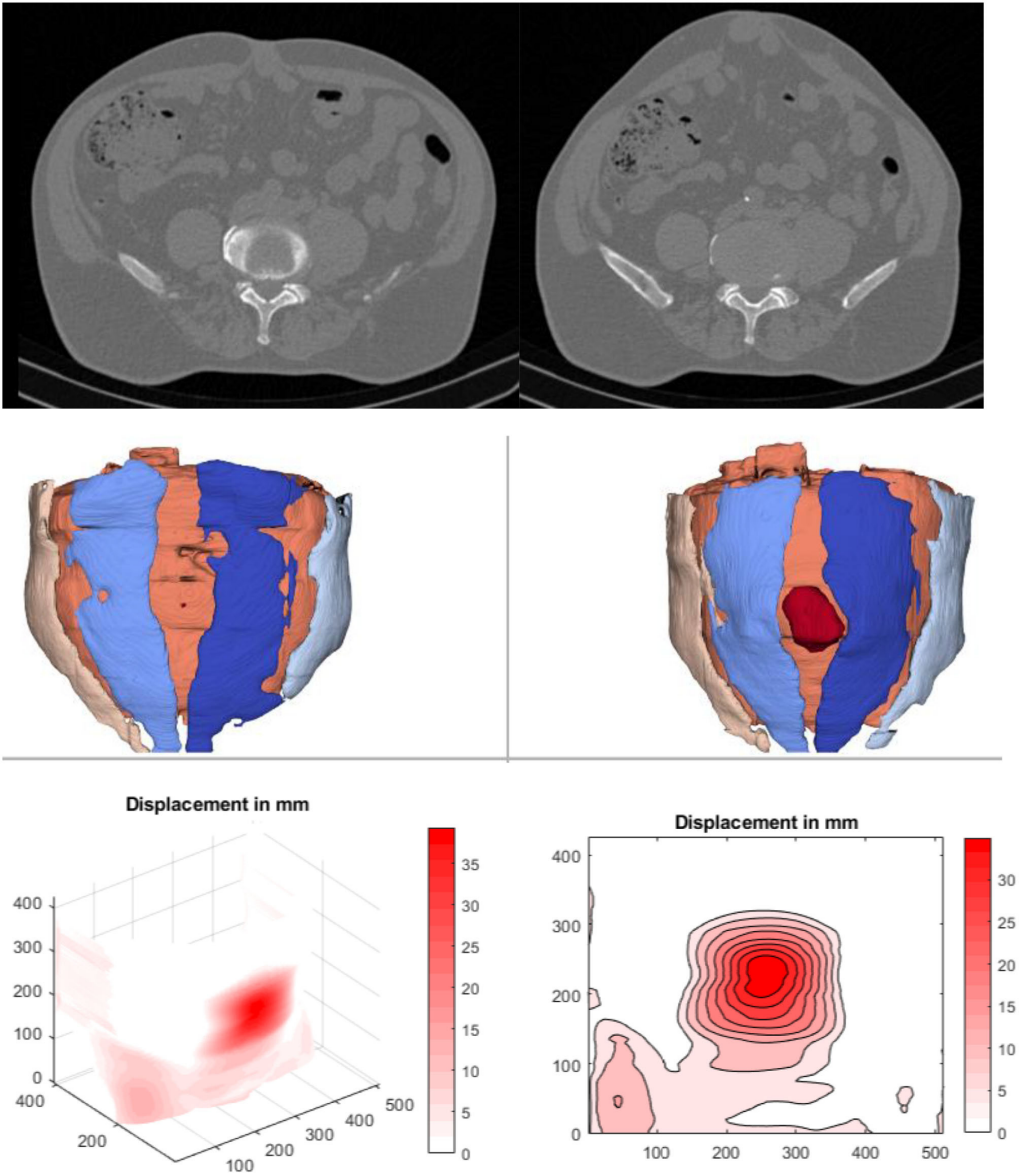
Imaging of a male, 50–55 years old, with an incisional hernia after an umbilical hernia repair. **Top:** A single slice of an abdominal CT scan at rest (left) und during a Valsalva maneuver (right) after the slice recognition of the DICOM data using 3D Slicer^®^. It is obvious that load deforms not only the median hernia but the entire abdominal wall. **Middle:** Result of the automatic segmentation using the artificial intelligence described above. At rest, the surface does not bulge due to a missing protrusion (left). Once the protrusion gets pronounced during a Valsalva's maneuver, the hernia becomes obvious (red volume in the right middle panel). The rectus musculature is depicted light and dark blue to the right and to the left of the hernia orifice. Between the musculature, the linea alba is lightly cream colored and thinned out to a plane. **Lower panel:** A surface projection of the tissue displacement upon Valsalva (left) and an isodistance mapping of the superficially visible displacement fields (right)—closely adjacent lines show high gradients and thus main stress zones. The center of the hernia opening in the evaluation is colored darkest red indicating a displacement of almost 4 cm.

CT scanning of the abdomen was performed using a low-dose protocol derived from kidney stone scans. The volume from the symphysis to the diaphram was scanned twice without contrast medium during deep inspiration. The CT data were collected sequentially with a slice thickness of 0.6 mm at 110–130 kV. The first series was acquired with the patient at rest. The second series required the patient to strain himself as hard as he could (Valsalva maneuver). The native DICOM files were imported into the 3D slicer image computing platform (3D Slicer image computing platform | 3D Slicer). Figure 1 gives an example of the result in the upper panel.

Hernia parameters were determined using Biomedisa (Biomedisa) und Matlab 2019b (The MathWorks Inc., Massachusetts, USA). Automatic segmentation was carried out with Biomedisa (16). A deep neural network (AI) was trained on 23 volumetric datasets that were manually segmented by a clinician. The datasets consist of 13 patients. In 10 patients, both the first series at rest and the second series under load (Valsalva maneuver) were used. Only one state was considered for the remaining three patients. The training process took about 12 h. Using the trained neural network, the automatic segmentation of additional volumetric images takes 21 s with a NVIDIA GeForce GTX 1080 Ti.

The second approach is based on non-rigid registration of the CT data at rest and during a Valsalva maneuver (17). The movement of the abdominal wall from one state to the other is captured and a local displacement is derived (18). The more compliant, unstable areas of the abdominal wall displace more under load.

The investigators measured an average hernia area of 51 cm^2^ at rest and 61 cm^2^ during the Valsalva maneuver manually using a previously published procedure (19). The AI-assisted segmentation resulted in a hernia area of <1 cm^2^ at rest and a hernia opening of 28 cm^2^ under Valsalva. The unstable abdominal wall between the rectus muscles is determined to be 89 cm^2^ at rest and 86 cm^2^ under Valsalva. It was noted that the automated evaluation determined the unstable area during the Valsalva maneuver with a deviation of 9% as compared to the intraoperative determination (94 cm^2^). The manual evaluation underestimated the unstable zone by ~30%. Surgical treatment was performed with a rectangular 20 × 30 cm Dynamesh^®^ Cicat mesh (CRIP: 62; MDAR: 6.4; 70 fixation points with Prolene^®^ 2-0; GRIP 227). According to the current state of knowledge, the safety margin of 3.7 times the GRIP compared to the required CRIP will result in a permanently secure repair in this case. The patient reports him returning to heavy work after 3 months with no pain and no bulge after 1 year.

### Patient Characteristics

A total of 163 patients were repaired (119 primary incisional hernias, 44 recurrences). One patient with a primary incisional hernia was excluded from the analysis due to metastatic urothelium carcinoma followed by death 3 months after the hernia reconstruction. The remaining patient's age ranged between 27 and 92 years (mean ± SD: 63 ± 13 years). Primary and recurrent hernia were not significantly different (*p* = 0.01778). Gender was equally distributed with 81 patients in each group. Most patients were ASA3 (*n* = 84) or ASA2 (*n* = 65) with 12 ASA1 and 1 ASA 4. Body height was 171 ± 10 cm (range: 151–199 cm), body weight was 83 ± 19 kg (range: 41–140 kg). The average BMI was 28.5 ± 5.5 (range: 16.8–54.7). Weight was not different between primary and recurrent hernia (*p* = 0.1031). A total of 164 risk factors were present in primary (mean: 1.4), 66 risk factors in recurrent hernias (mean 1.5). Most patients had multiple incisions with 227 previous surgeries in primary hernias (mean: 1.9) and 112 procedures in recurrences (average: 2.5).

### Statistical Evaluation

The data were extracted from the Herniamed^®^/Stronghold registry and accumulated into Excel^®^ spreadsheets. Descriptive statistics were calculated using the inbuilt statistical features. The results were depicted as histograms for primary and recurrent incisional hernias. Differences were analyzed with open-source online programs [Mann-Whitney U Test Calculator (socscistatistics.com)].

## Results

### CT Abdomen at Rest and During Valsalva's Maneuver

**Figure 2:**
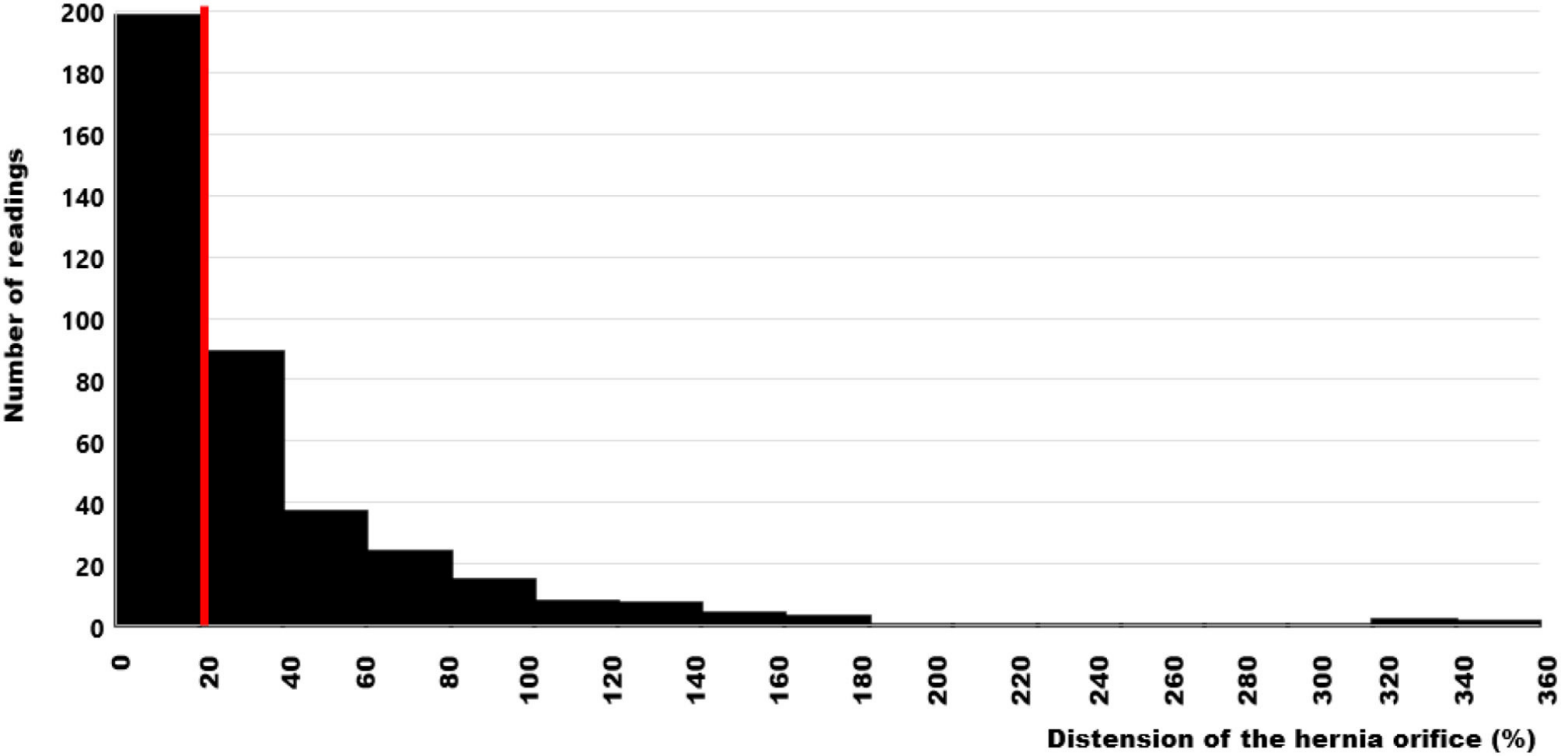
Histogram of the distension values of the hernia orifices during Valsalva's maneuver as compared with the hernia sizes at rest in 123 patients. The median value is indicated by the red bar. The data are truncated at 360% for clarity but reach 1800% as an extreme value.

Patients with highly unstable abdominal walls upon clinical examination were subjected to CT scans of the abdomen at rest and during a Valsalva maneuver according to a previously published protocol (19). A total of 1.164 readings of the distension of the hernia orifice upon pressure were determined from 123 patients with at least three different observers evaluating the hernia sizes at least four times each. The median distension of the hernia orifice was found to be 20% with a range from zero to 1800% (Figure 2). A distension of 20% was assumed for the forty patients clinically judged to have stable abdominal walls when their repair was designed from CT scans of the abdomen without straining for the CRIP calculation.

### Biomechanical Characteristics of the Repairs of Primary and Recurrent Incisional Hernias

**Figure 3:**
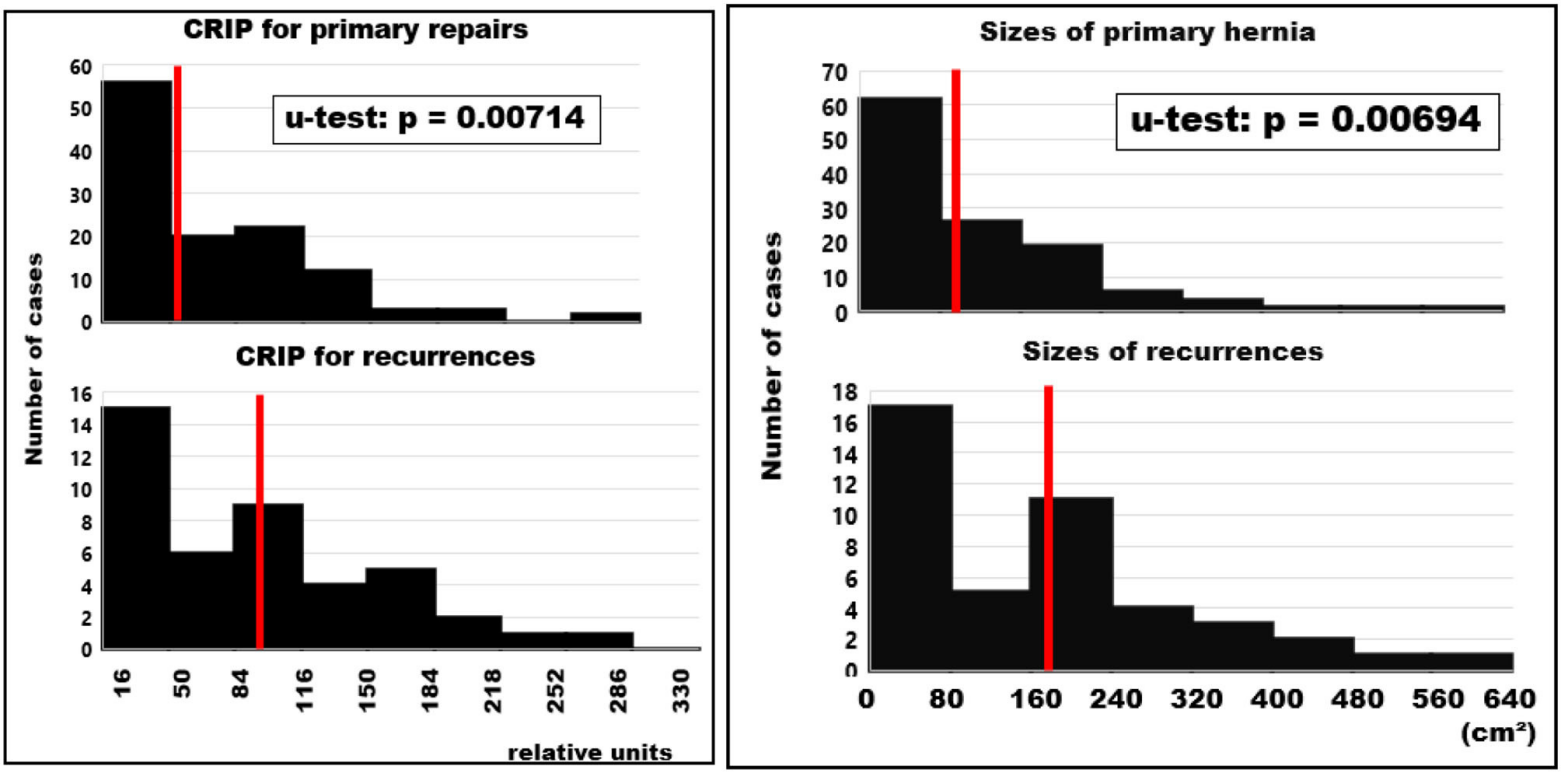
Frequency distributions of biomechanical characteristics used for the design of incisional hernia repair. The median values are indicated by red bars. **Left:** CRIP values for primary repairs and recurrent hernias taking into account the measured tissue distension calculated preoperatively. In cases with CT scans of the abdomen at rest only, a distension value of 20% was assumed. The median CRIP values of recurrent hernias are twice those calculated for primary cases (*p* = 0.00714). **Right:** Sizes of primary and recurrent incisional hernias taken as the larger area for incisional hernia repair. The sizes to be repaired for battlefield abdominal walls with multiple incisions and larger areas of instability can expand beyond the facial defects. Sizes of recurrent hernias were twice as large as primary defects (*p* = 0.00694).

From the preoperative measurements, the critical resistance of the repair toward dynamic impacts (CRIP) was calculated (Figure 3, left panel). Intraoperatively, the hernia sizes were measured after dissection and compared with the values derived from preoperative imaging. The larger values were taken as the area of instability to be augmented. These values were significantly different between primary and recurrent hernias. This finding is concomitant with the differences in the CRIP values between primary hernias and recurrences (Figure 3, right panel).

**Figure 4:**
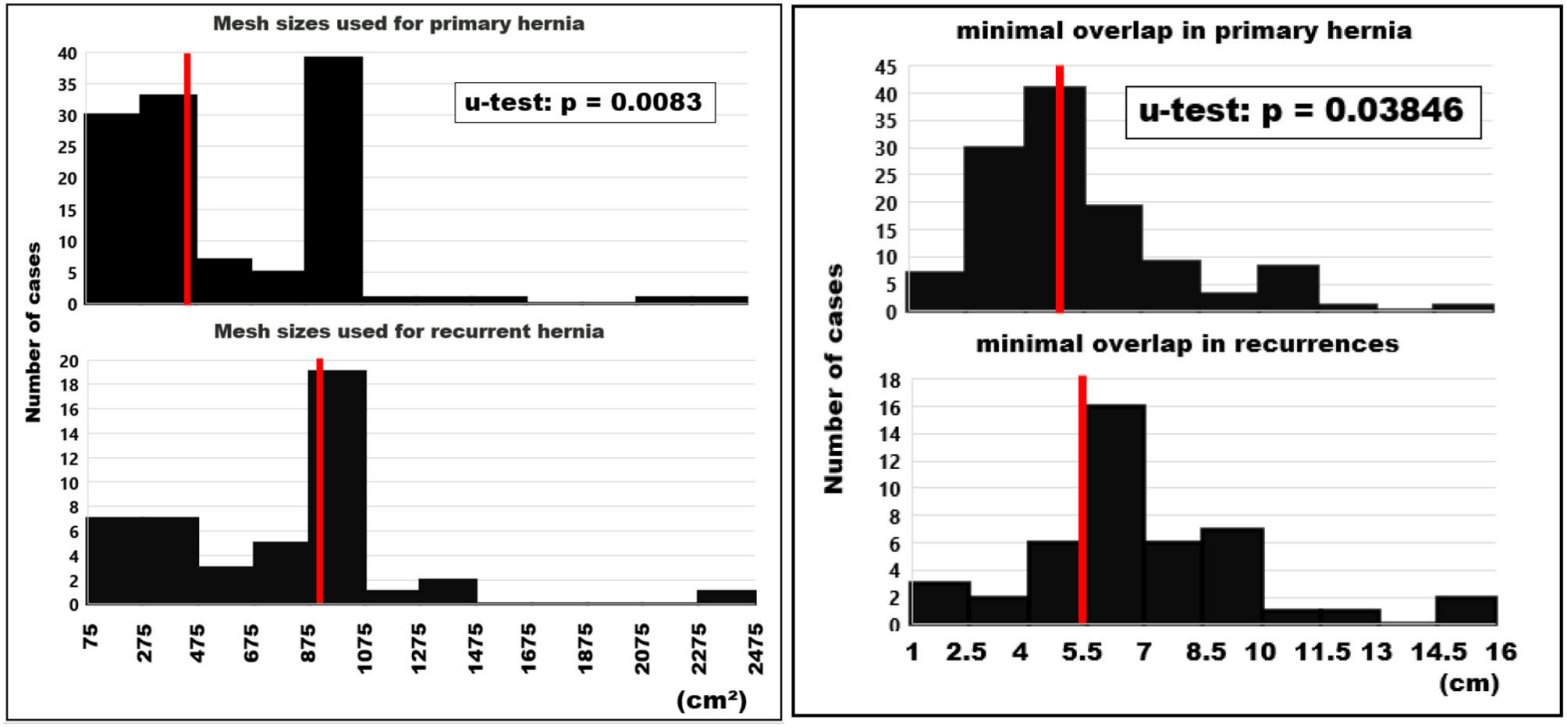
Histograms of the mesh sizes **(left)** and the minimal mesh overlap **(right)** of primary and recurrent hernias. The median values are indicated by red bars.

Since the reconstructions needed to surpass the CRIP values to be considered durable repairs, mesh sizes were significantly larger in recurrent compared to primary hernias. Minimal overlap was slightly smaller in primary as compared to recurrent hernias giving more strength to more unstable abdominal walls (Figure 4). Based on empirical observations, the textile should overlap the border of the unstable area of the abdominal wall by at least 50 mm. This limit is surpassed by half the primary reconstructions and 54% of the recurrent repairs. In general, notch effects were neglected and the meshes were oriented longitudinally along the axis with the highest elasticity.

**Figure 5:**
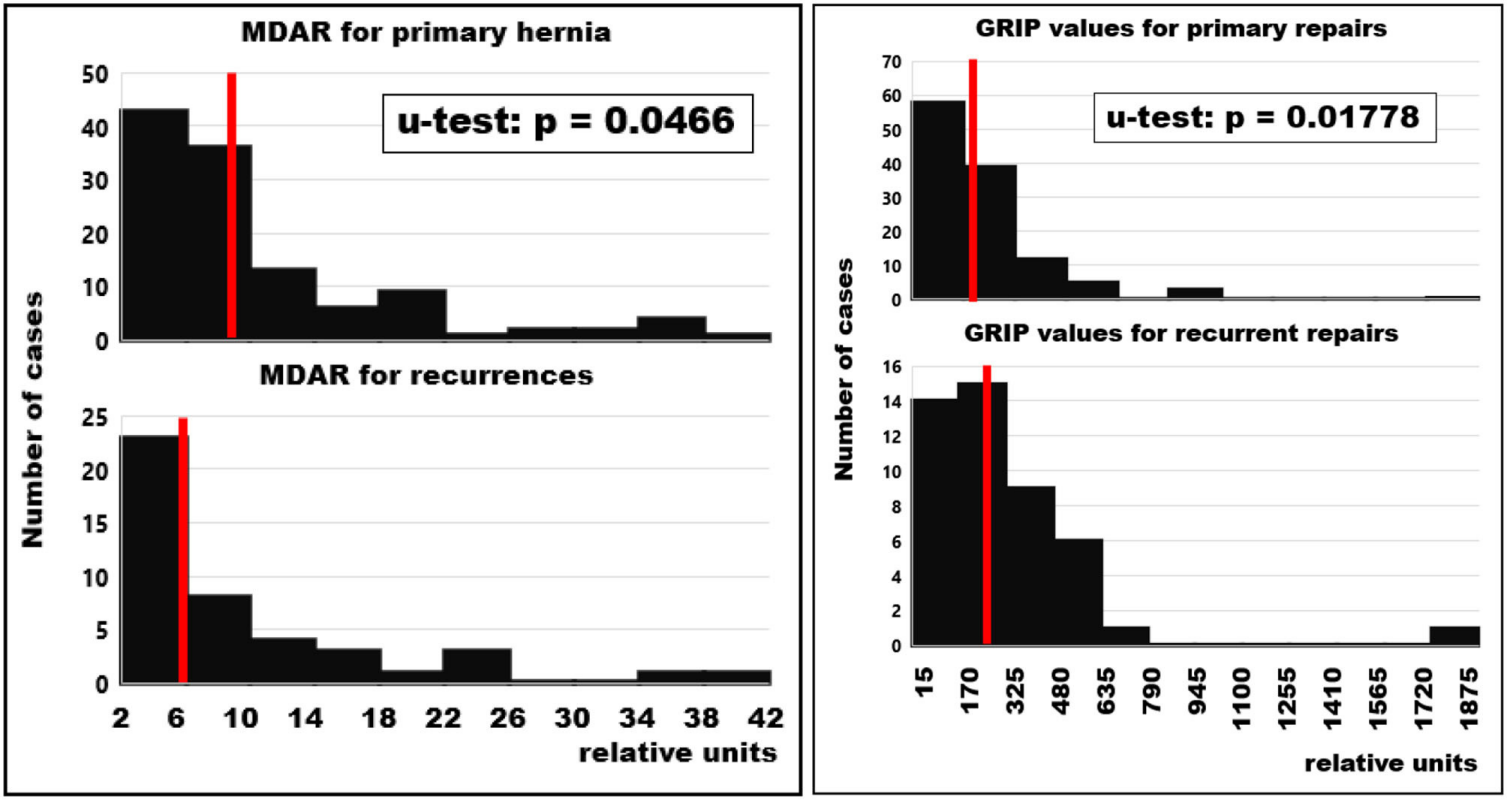
Frequency distributions of the mesh-defect-area ratio (MDAR) for primary and recurrent hernias **(left)** and gained resistances toward impacts related to pressure (GRIP) after the repair of primary and recurrent hernias **(right)**. Red bars indicate median values.

The mesh-defect-area ratio (MDAR) describes the relationship between reinforcement and defect in hernia repair. MDAR is higher in primary than in recurrent incisional hernias (Figure 5). The binding effect of fixation elements was used to compensate the reduced dynamic stiction due to lower MDAR values. In primary hernias, a median of 42 fixation points was used. In 73 patients, it was sutures (mainly Prolene^®^ 2-0), in 41 sutures and tacks, in two sutures, tacks and glue and in one only tacks. Unsecured mesh was used in one patient (Progrip^®^ 225 cm^2^ after an e-retro transversus abdominis release with laparoscopic Vloc^®^ closure of a 3 × 6 cm defect). In recurrent cases, a median of 84 fixation points was applied. Sutures (Prolene^®^ 2-0 with one exception) were combined with tacks in 24 repairs. Only sutures were used in 18 reconstructions. Sutures plus tacks plus glue was used in one case. In another patient, sutures were combined with 2 ml of fibrin glue applied to 20 cm^2^. All defects were closed using peritoneal flaps in 29 recurrences and 41 primary repairs to achieve low abdominal tension. In all cases, intraperitoneal pressure was monitored via an indwelling catheter, which was removed once the intraabdominal pressure was below 20 cm ${\text{H}}_{2}\text{O}$ postoperatively.

**Figure 6:**
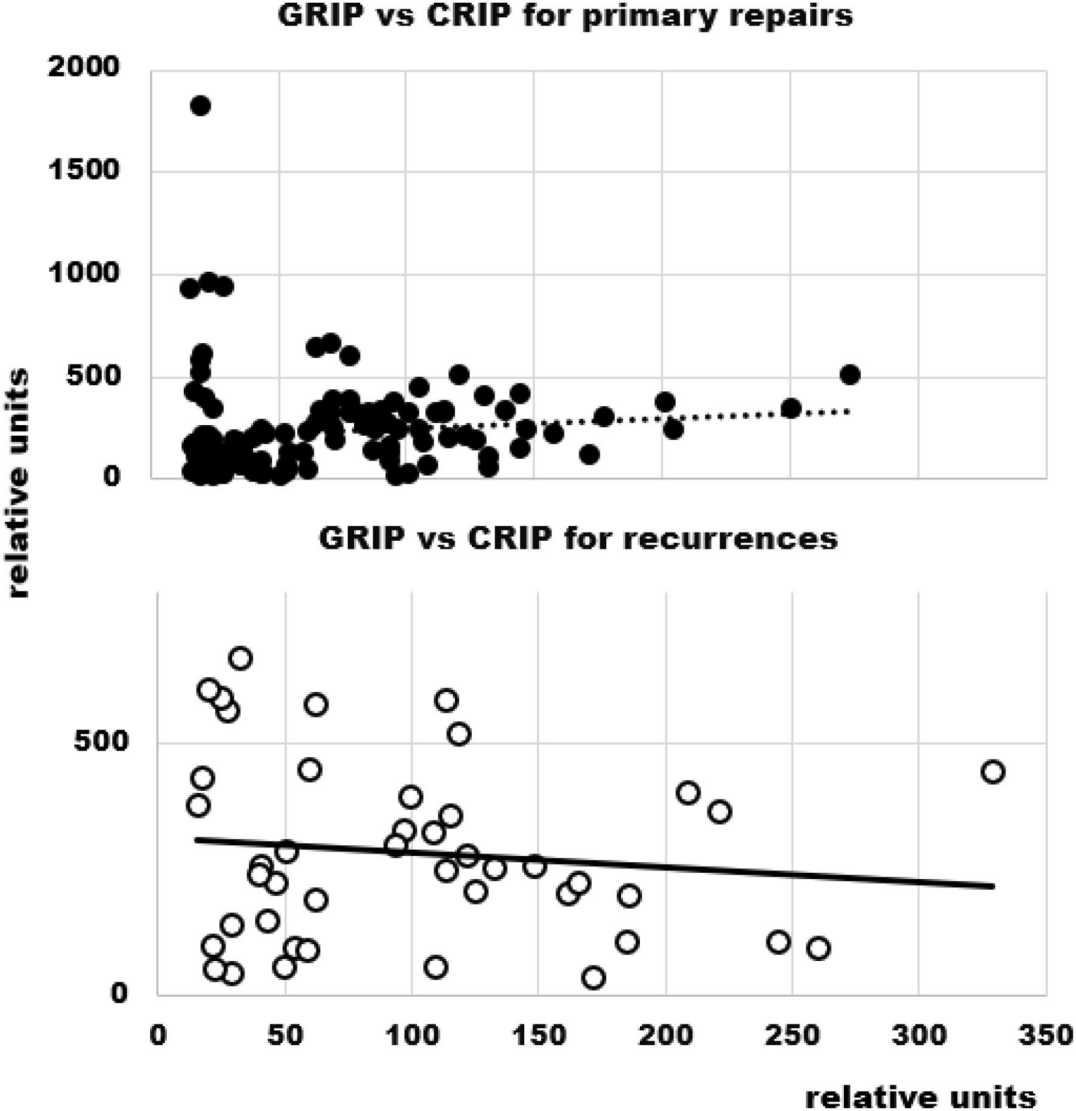
GRIP as a function of CRIP in primary and recurrent hernia repair. The dashed **(top)** and the solid line **(bottom)** indicate the trend.

The GRIP values were lower in primary hernias (Figure 5). This reflects the larger hernia sizes in redo operations and the subsequently elevated CRIP values. Considering GRIP as a function of CRIP, it rises in primary repairs and tends to drop in recurrent hernia repair (Figure 6). In both, the correlation coefficient is 0.1 and can only be regarded as a trend. Very high GRIP values at very low CRIP levels required indicate potential to save valuable OR time in the future.

### Surgical Characteristics of the Repairs of Primary and Recurrent Incisional Hernias

**Figure 7:**
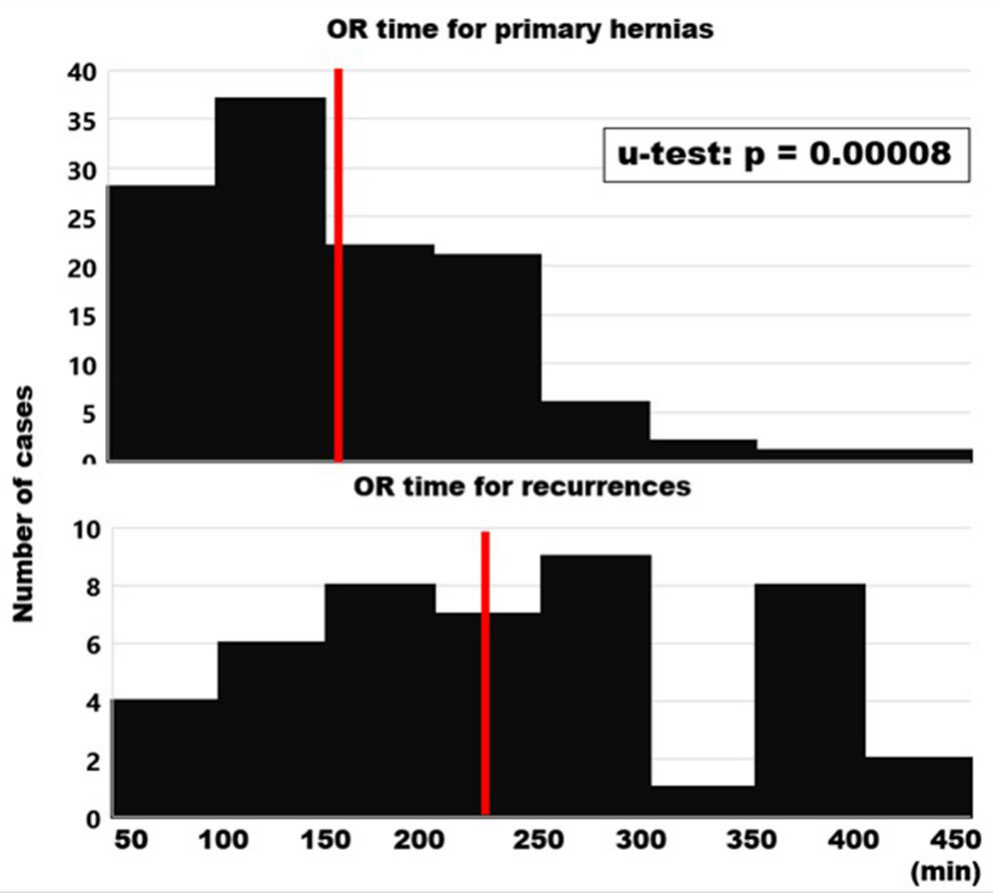
Histograms of the incision-to-closure times. Red bars indicate the median values.

The incision-to-closure time gives an indication of the effort necessary to achieve biomechanically stable repairs (Figure 7). The median for primary hernias was 150 min (mean ± SD; range: 160 ± 76; 47–480 min). Redo surgery took much longer with a median of 228 min (225 + 89; 50–420 min). The longest primary case was a hernia of 259 cm^2^ including a parastomal repair with a body mass index (BMI) of 38, four previous open surgeries including aortic and colorectal interventions, a diffuse bleeding disorder and a chronic obstructive airways disease. The longest recurrent case was 6 years after a desmoid tumor resection of the abdominal wall with an open abdomen closed with a Proceed^®^ mesh, an underlying coagulation deficit and a hernia opening of 412 cm^2^. Both cases were closed with a 1060 cm^2^ Dynamesh^®^ CiCAT mesh.

**Figure 8:**
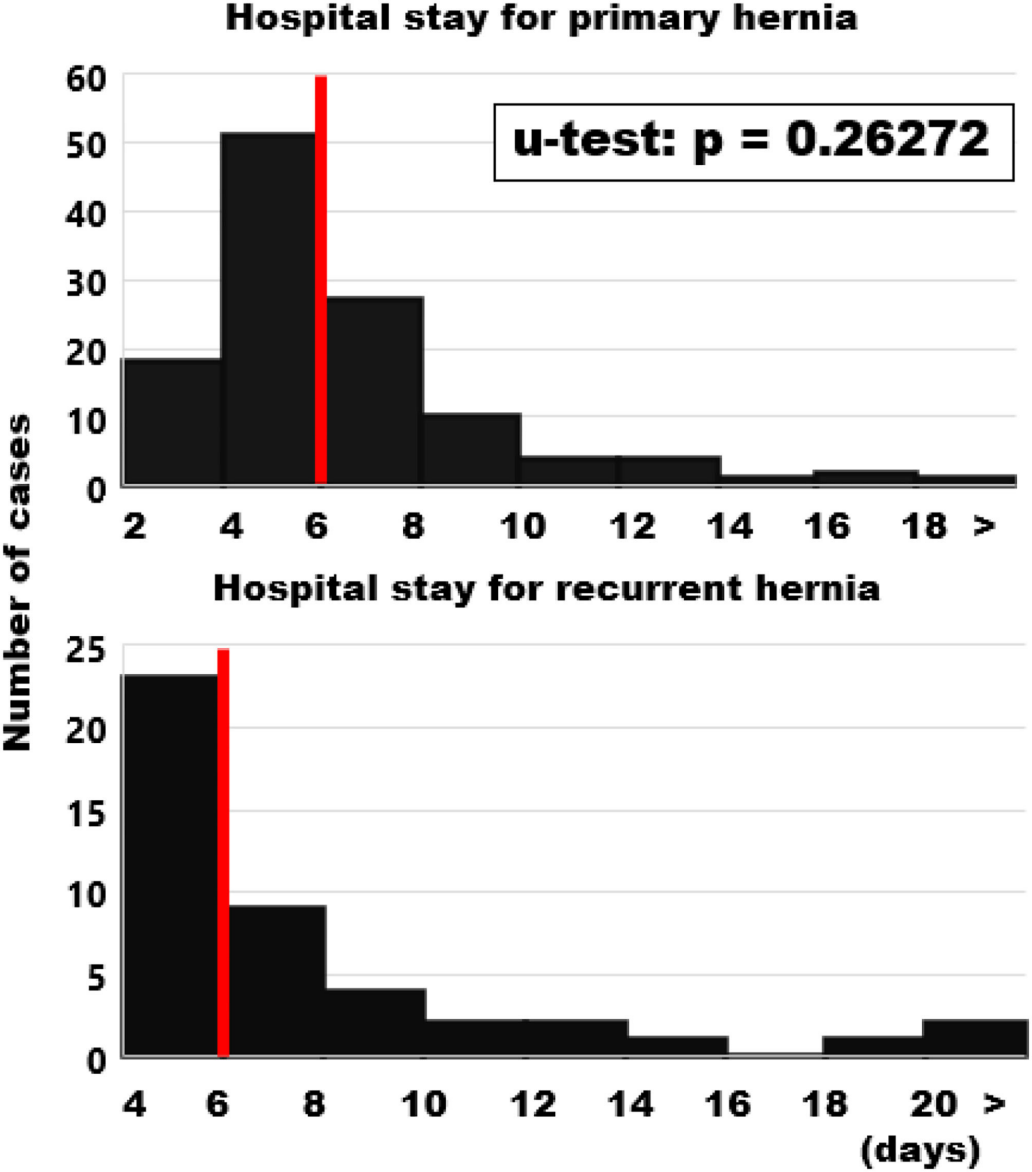
Frequency distributions of the length of hospital stay. Please note the shift of 2 days in the ordinate. Median values are indicated by red bars.

Half of the patients in both groups were discharged after 6 days (Figure 8). Patients with large hernias requiring a component separation, peritoneal flaps, sandwich techniques with additional biosynthetic onlay or underlay reconstructions, omental or falciforme flaps and local advancement flaps required up to 2 weeks for recovery. Ten patients with primary reconstructions had a prolonged stay due to seroma formation (5), pneumonia (3), pulmonary embolism (2), postoperative bleeding (1) and delayed wound closure (1). In recurrent cases, five patients had delayed wound healing (3), seroma formation (1), urinary tract infection with tumor blocking the one remaining, stented ureter (1) and pulmonary embolism (1). The patient with the longest stay (113 days) had a BMI of 37, chronic obstructive airway disease requiring steroids, a diffuse bleeding disorder, the fourth recurrence and reactivated a previously acquired methicillin-resistant staphylococcus aureus in the secondary healing wound. No patient died in hospital or within 1 month after discharge.

**Figure 9:**
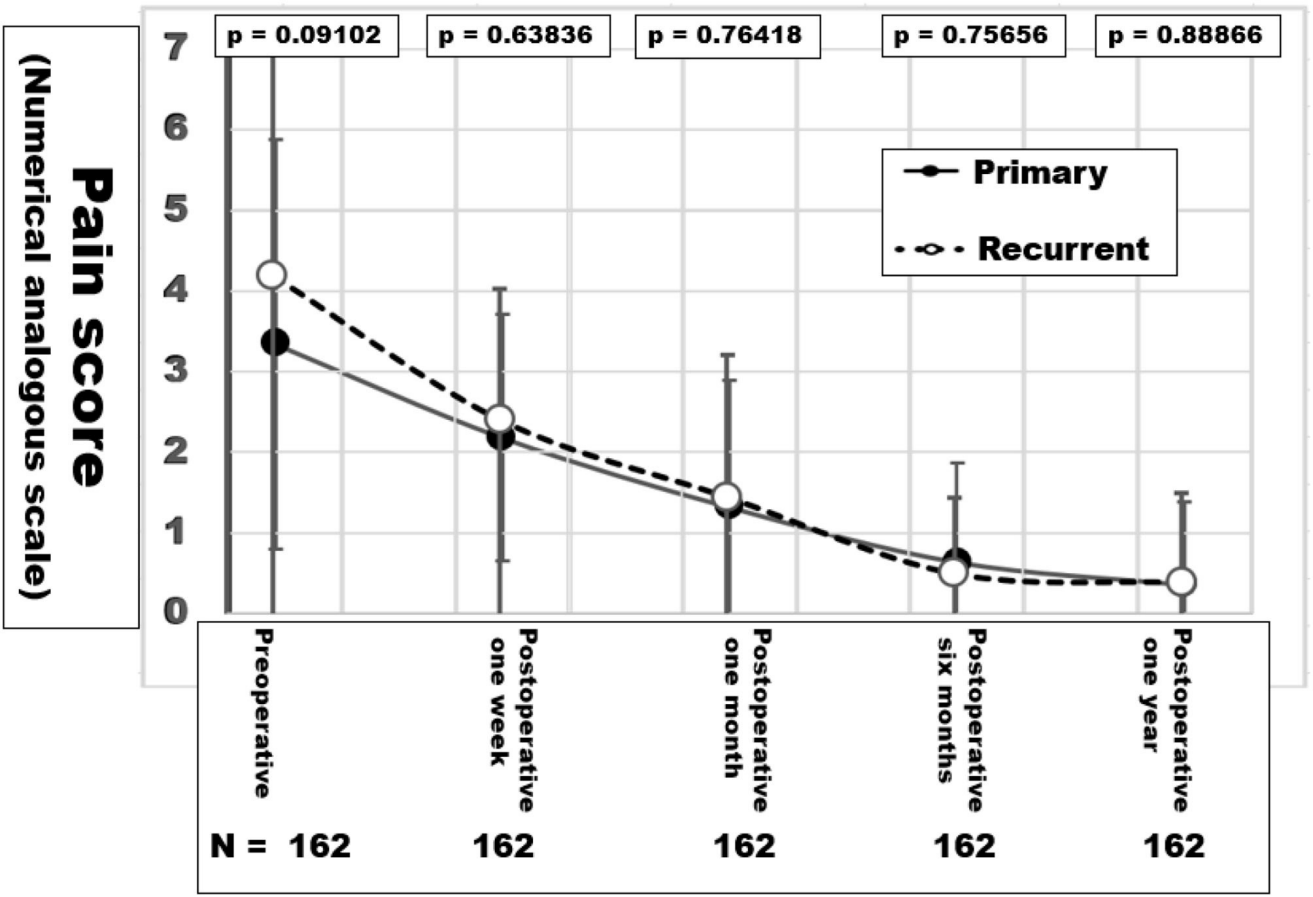
Pain levels in NAS values during the first year of follow-up after hernia repair. The *p*-values in the inset indicate similar pain levels after the abdominal wall reconstruction in both groups.

Upon follow up for 1 year, no infield recurrence was observed (Figure 9). In both primary and recurrent hernia repairs, pain subsided without differences between the groups. All patients below the age of 62 regained their previous activities with full integration into the work process. Six patients gained weight up to 25 kg (five primary, one recurrent) and developed inguinal hernia (2), epigastric hernia in a rectal diastasis and incisional hernia in an intercurrent appendectomy and a cholecystectomy scar (one each). One infected seroma developed 3 months after discharge and required surgery with the preservation of the mesh so far. One patient with a primary hernia repair had an explorative laparotomy for pain and suspected hernia despite lacking evidence on a preoperative CT scan with the intraoperative finding of an endometriosis and five subsequent explorations for endometriosis without a recurrent hernia so far.

## Discussion

Mesh-related complications are claimed frequently since recurrence and pain are common after incisional hernia repair. As of July 15, 2021, there were more than 20,000 mesh-related lawsuits pending in federal and state courts across the USA (https://www.drugwatch.com/hernia-mesh/lawsuits/). Considering biomechanics, recurrence and pain reflect a lack of mechanical strength at the mesh-tissue interface. Current research is directed toward the early identification of risk groups to minimize recurrence rates, pain and misery for the patients and high costs for the society (20). The biomechanical approach defines a load limit. Cyclic loading allows a new view on the development and repair of incisional hernia. Each abdominal wall has its unique biomechanics which can be elucidated by CT abdomen with Valsalva (Figure 1). The distention of the abdominal wall of the individual patient can be assessed preoperatively (Figure 2). Bench testing with cyclic loads can characterize components used for the reconstruction of the abdominal wall. Such characterizations should be on every package label to support the surgical work (10). The design of incisional hernia repair can consider all influences known so far (Figures 3–8). Effects on resource consumption and management can be assessed potentially saving pain for the patients and money for public healthcare by reducing operating times at low recurrence rates. Pain levels subside rapidly even after large reconstructions based on biomechanical considerations (Figure 9). Patients return to work and avoid old-age poverty.

Complications may be falsely attributed to meshes if a potential mismatch of the mechanical properties of the meshes as part of the repair and the loads acting on them is ignored (12). Lawsuits, public criticism, more demanding administrative and governmental regulations and reduced availability of hernia meshes may result (21). According to internet sources, more many thousand mesh-related law suits are still pending in 2021 after the settlement of roughly 3,000 cases for 184 million US$ (www.drugwatch.com/hernia-mesh/lawsuits/). Biomechanically stable repairs may require an additional 180 million US$ at the first approach due to longer OR time and more material use. Calculating from social and law data partially given above, society can save 1.8 billion at the same time avoiding recurrences, pain, unemployment and old-age poverty.

The concept of resistance against pulse load is common in technical engineering, material science and polymeric compound design. Shakedown or breakdown mechanisms of tissues connected to textiles depend on small changes in the compound structure which we have just started to explore (22–24). Spreading of cells, fiber formation and growth of soft tissues require cyclic stretching (25). Freshly formed collagen fiber networks cannot bear load but stretch upon repeated pulse load (2). Once the load limit is surpassed, a rapid breakdown of an incisional hernia repair is observed (26). The minimal requirements for biomechanical stability can be derived from a cyclic load bench test, expressed in coefficients and calculated before (CRIP), during and after surgery (GRIP). Adding GRIP values to a registry permits comparative analysis of groups of patients with well-defined end points (the Herniamed^®^/Stronghold registry Die Herniamed-Studie und die Stronghold-Studie–Hernien-Selbsthilfe Deutschland e. V.).

Notch effects and stress concentrations are not yet integrated into the biomechanical concept but will need to be evaluated starting with the suturing of incisions and defects. Where is the limit of suturing? When do we need prophylactic meshes? Can our techniques be performed flawlessly? Potential interactions between meshes, fixation elements, closure techniques and failure mechanisms can be analyzed on a bench test with cyclic loading. In the future, bench test results can be transferred to the clinical application. The evaluation in a prospective observational registry paves the way to randomized studies with well-defined biomechanical goals. This approach enables future progress.

**Figure 10:**
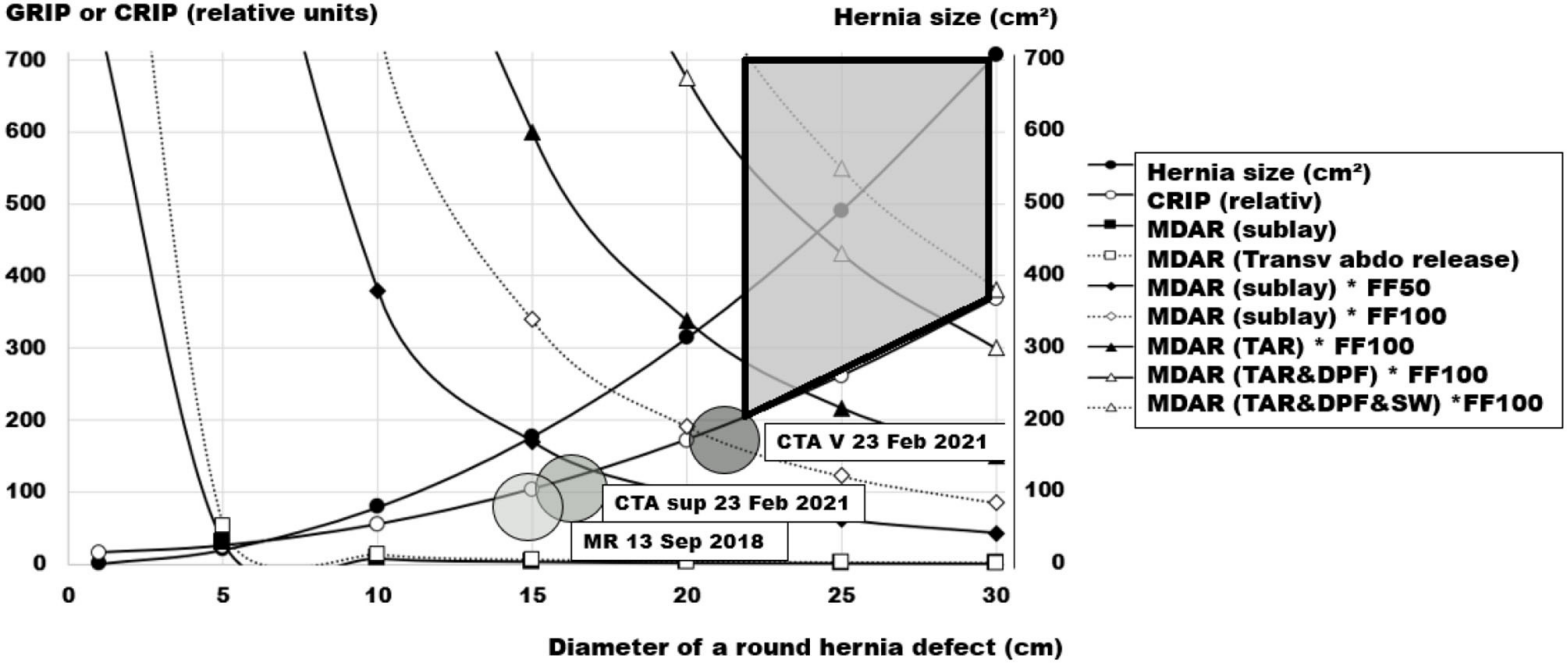
Pre-operative assessment of the incisional hernia of a liver transplant recipient, 65–70 years old, as a function of the diameter of his round hernia defect. The large shaded circles indicate the hernia sizes at various time points with magnetic resonance (MR), computed tomography of the abdomen (CTA) at rest (sup) and during a Valsalva maneuver (V). The required critical resistance to impacts related to pressure (CRIP) is depicted by the small circles, the hernia size at a given diameter by the dots. The cornered symbols indicate the respective resistance toward impacts related to pressure (GRIP) gained by a 30 × 45 cm ellipsoid mesh with a coefficient of 1 in various mesh-defect area ratios (MDAR), a sublay/retromuscular position, combined with a posterior component separation (TAR), and two distinct fixation factors (FF50 = 100 suture points or FF100 = 200 sutures points). A double peritoneal flap reconstruction (DPF) gains space for mesh placement. A sandwich reconstruction (SW) with a biosynthetic mesh adds to the MDAR of the reconstruction. The area covering durable repair options is shaded in light gray with marked borders.

The clinical course of individual patients can be considered, at the same time taking into account repair options. An example is given in Figure 10 which depicts the enlargement of the incisional hernia of a liver transplant recipient and the various options for reconstruction in this case. From these data, a reconstructive approach was designed.

In this way, the value of different options for the reconstruction of the individual patient can be visualized. Adequate measures can be refined further at the same time avoiding overtreatment. For the patient under consideration, a posterior component separation for the placement of a 30 × 45 cm mesh with a coefficient of at least one should be combined with 200 sutured stitches. A combination with flaps or a sandwich reconstruction is not necessary in this case. Considering the treatment options more generally, small hernias can be durably repaired by more inexpensive options. Larger hernia openings require advanced, extensive and resource-consuming procedures for a biomechanically stable repair. Since the cost of recurrence may be high in these cases, reimbursement schemes should allow for a better repair at the first approach.

After 1 year, both primary and recurrent patients hat no recurrence and low pain levels in our study group. Using a generalized linear model to compare different treatment options, Deerenberg et al. (27) compared different repair regimes. Open reconstructions without mesh had a recurrence hazard per year of 6.3%. Open repairs with mesh recurred on the average in 1.6%. Laparoscopically treated cases recurred in 3.1%. Our results compare favorably after 1 year. Future studies need a longer follow-up.

## Conclusions

Biomechanically stable repairs can be designed and adapted to the patients' needs with the GRIP concept. The clinical application results in complication rates below 10% in both primary and recurrent incisional hernia. After 1 year, low pain levels and no recurrences were observed in 162 consecutive cases treated according to this concept in four hospitals by ten surgeons.

## Data Availability Statement

The raw data supporting the conclusions of this article will be made available by the authors, without undue reservation.

## Ethics Statement

The studies involving human participants were reviewed and approved by Ethikkommission der Medizinischen Fakultät Heidelberg S-522/2020. The patients/participants provided their written informed consent to participate in this study.

## Author Contributions

RN and FK designed the research, got the funding, analyzed the data, and drafted the manuscript. RN, TL, JR, and FK designed the clinical incisional hernia repair, performed and/or supervised the surgery, and collected the data. FK, RN, TL, PL, VH, SV, and JG planned, performed, and evaluated the CT scans of the abdomen with Valsalva's maneuver. Y-ML and LA-H assisted in the collection of data and performed and/or supervised the clinical follow-up. All authors critically revised and finally approved the manuscript.

## Funding

This study was supported by Heidelberger Stiftung Chirurgie Grant Nos. 2016/22, 2017/171, 2018/215, 2019/288, 2020/376, and 2021/444.

## Conflict of Interest

FK has received research grants from Baxter^®^, Dahlhausen^®^, and Medtronic^®^ not related to the research perspective described in the manuscript. The remaining authors declare that the research was conducted in the absence of any commercial or financial relationships that could be construed as a potential conflict of interest.

## Publisher's Note

All claims expressed in this article are solely those of the authors and do not necessarily represent those of their affiliated organizations, or those of the publisher, the editors and the reviewers. Any product that may be evaluated in this article, or claim that may be made by its manufacturer, is not guaranteed or endorsed by the publisher.
